# Supplementary material for: Complexome profiling of the Chlamydomonas psb28 mutant reveals TEF5 as an early PSII assembly factor
Source: Plant Cell. 2025 Mar 17;37(6):koaf055. doi: 10.1093/plcell/koaf055 (PMC12164590; doi:10.1093/plcell/koaf055)
Supplement: koaf055_Supplementary_Data [file koaf055_supplementary_data.zip › psb28_interactive.zip!/plots/intensity/qualityPlots/peptides/GWD2_Cre07.g332300.t1.2.html]

### Cre07.g332300.t1.2

Trivial name: GWD2  
SeqCoverage [median]: 0.90  
Mass: 124.09 kDa  
  
 

| Sequence | MissedCleavage | Mass [Da] | wtIntensity | muIntensity | Score |
| --- | --- | --- | --- | --- | --- |
| WTMYDILGHR | 0 | 1290.62 | 76092.83 | 50150.26 | 55.06 |
|  |  |  | 76092.8 | 50150.3 |  |
